# Synergistic inhibition of colon cancer growth by the combination of methylglyoxal and silencing of glyoxalase I mediated by the STAT1 pathway

**DOI:** 10.18632/oncotarget.18601

**Published:** 2017-06-22

**Authors:** Yuan Chen, Lei Fang, Gefei Li, Jiali Zhang, Changxi Li, Mengni Ma, Chen Guan, Fumao Bai, Jianxin Lyu, Qing H. Meng

**Affiliations:** ^1^ Key Laboratory of Laboratory Medicine, Ministry of Education of China, Zhejiang Provincial Key Laboratory of Medical Genetics, School of Laboratory Medicine and Life Sciences, Wenzhou Medical University, Wenzhou, Zhejiang 325035, China; ^2^ Department of Laboratory Medicine, The University of Texas MD Anderson Cancer Center, Houston, TX 77030, USA

**Keywords:** colon cancer, methylglyoxal, glyoxalase I, synergistic inhibition, STAT1

## Abstract

Methylglyoxal (MG), an extremely reactive glucose metabolite, exhibits antitumor activity. Glyoxalase I (GLOI), which catalyzes MG metabolism, is associated with the progression of human malignancies. While the roles of MG or GLOI have been demonstrated in some types of cancer, their effects in colon cancer and the mechanisms underlying these effects remain largely unknown. For this study, MG and GLOI levels were manipulated in colon cancer cells and the effects on their viability, proliferation, apoptosis, migration, and invasion *in vitro* were quantified by Cell Counting Kit-8, colony formation assay, flow cytometry, and transwell assays. The expression levels of STAT1 pathway–associated proteins and mRNAs in these cells were quantified by western blot and qRT-PCR, respectively. The antitumor effects of MG and silencing of GLOI were investigated *in vivo* in a SW620 colon cancer xenograft model in BALB/c nude mice. Our findings demonstrate that MG in combination with silencing of GLOI synergistically inhibited the cancer cells’ proliferation, colony formation, migration, and invasion and induced apoptosis *in vitro* compared with the controls. Furthermore, these treatments up-regulated STAT1 and Bax while down-regulating Bcl-2 *in vitro*. MG treatment alone or in combination with silencing of GLOI also reduced the growth of the SW620 tumors in mice by up-regulation of STAT1 and Bax and down-regulation of Bcl-2. Taken together, our findings suggest that MG in combination with silencing of GLOI merits further evaluation as a targeted therapeutic strategy for colon cancer.

## INTRODUCTION

Colon cancer is the third most common cancer and the third most common cause of cancer-related death worldwide [1]. In China, it is the fourth most common cancer and the fifth leading cause of mortality among cancer patients, both men and women [2]. At least 50% of patients with colon cancer develop metastases, most of which are unresectable [3]. The main treatment options for colon cancer are surgery and chemotherapy, and their efficacy depends on the cancer stage and tumor location at diagnosis as well as individual patient characteristics [4]. Although much work has been done to refine these therapies, the high frequency of drug resistance and tumor metastasis limit their clinical efficacy [5–7]. Therefore, new and more effective therapeutic approaches are profoundly needed.

Methylglyoxal (MG), an extremely reactive glucose metabolite, is an endogenous byproduct of glycolysis. It is mainly generated in the dephosphorylation of glycolytic intermediates and in metabolism of the polyol pathway and amino acetone [8–13]. MG was historically investigated as an antitumor agent, but sound scientific evidence of its efficacy is lacking [9–16]. Previous studies from our laboratory showed that MG suppressed human colon cancer cell lines and tumor growth in a mouse model and had inhibitory effects against breast cancer [12, 13]. MG has been reported to prevent tumor growth by inhibiting mitochondrial respiration in malignant cells [17].

The glyoxalase system is a ubiquitous detoxification pathway that protects against cellular damage caused by potent cytotoxic metabolites, such as MG. It consists of glyoxalase I (GLOI) and GLOII. GLOI, using reduced glutathione as a co-substrate, converts MG to S-D-lactoylglutathione, which is further hydrolyzed by GLOII to D-lactate [18–21]. Ranganathan et al. demonstrated that GLOI expression was greater in human colon cancers than in normal tissue from the same individuals [22]. Overexpression of GLOI has also been documented in tumor cells, including colon cancer cells [19–26]. Tumor cells became more sensitive to cell killing agents when their GLOI expression was down-regulated [21].

Signal transducer and activator of transcription-1 (STAT1), a member of the STAT family, is a tumor suppressor, preventing development and progression of established tumors [27, 28]. A previous study indicated that STAT1 could inhibit the growth of neoplastic cells by regulating caspases, BCL-xL, and p21waf [27]. Antonis et al. demonstrated that STAT1 is a bona fide suppressor of breast tumorigenesis [29]. Another study has demonstrated that STAT1 may be a negative regulator of the development and progression of human hepatocellular carcinoma through induction of apoptosis and cell cycle arrest [30]. Jonathan M et al. suggested that hyperplastic polyposis 1s (HPP1s) tumor-suppressive activity is mediated at least in part by up-regulation STAT1 in colon cancer cells [31].

Although ample evidence supports the association of MG and the glyoxalase system with suppression of tumorigenesis, the role of MG, especially in combination with the glyoxalase pathway, in colon cancer and the mechanisms underlying that role are not clearly illustrated. In this study, we investigated the anticancer effects of MG alone or in combination with silencing of GLOI and their potential underlying molecular mechanisms, particularly with involvement of STAT1 pathway in colon cancer using cell lines and an animal model.

## RESULTS

### Expression of GLOI in colon cancer cells

**Figure 1:**
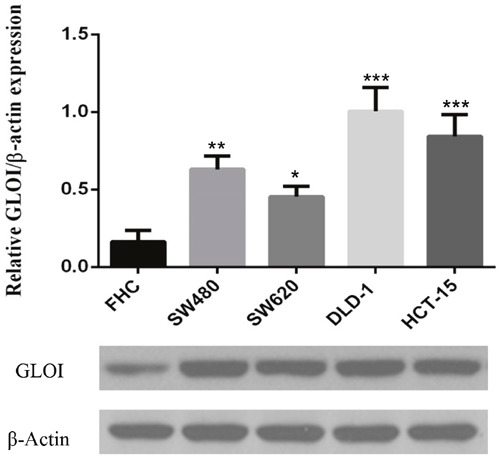
GLOI is overexpressed in colon cancer cells Expression of GLOI was determined by Western blotting in normal colon cells (FHC) and in four colon cancer cell lines: SW480, SW620, DLD-1, and HCT-15. β-Actin was used as the internal control. *p < 0.05, ^*^p < 0.01, ^**^*p < 0.001 vs shNC. All data are representative of three independent experiments (n = 3). Differences between groups were analyzed by analysis of variance.

GLOI expression was significantly greater in SW480 (*p* < 0.01), SW620 (*p* < 0.05), DLD-1(*p* < 0.001), and HCT-15 (*p* < 0.001) colon cancer cells than in normal colon FHC cells (Figure 1). The level of GLOI protein varied from 3-fold to 8-fold greater in the colon cancer cells than in the normal cell.

**Figure 2:**
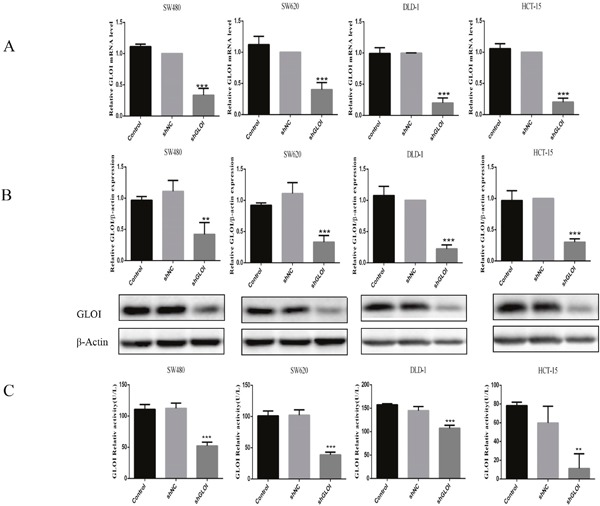
GLOI silencing reduces GLOI mRNA and protein levels and enzyme activity in colon cancer cells Colon cancer cells were transfected with shGLOI, empty vector (shNC, negative control), or nothing (no transfection) (Control). The expression levels of *GLOI* mRNA **(A)** and GLOI protein **(B)** in the cells were determined, as was GLOI enzyme activity **(C)**. ^*^p < 0.01, ^**^*p < 0.001 vs shNC. All data are representative of three independent experiments (n = 3). Differences between two groups were analyzed by analysis of 2-tailed Student t-test.

The expression levels of GLOI protein and mRNA were significantly lower in colon cancer cells transfected with the GLOI shRNA targeting sequence (shGLOI) than in cells transfected with a empty vector construct (shNC; *p* < 0.01 to 0.001; Figure 2A and 2B). Similarly, GLOI enzyme activity was significantly lower in the colon cancer cells transfected with shGLOI than in the shNC group (*p* < 0.01 to 0.001; Figure 2C).

### MG, alone or in combination with GLOI silencing, inhibited viability and proliferation of colon cancer cells

**Figure 3:**
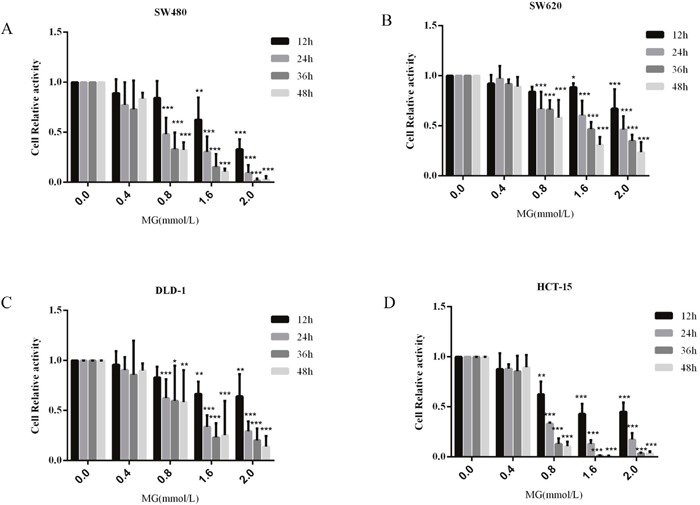
Methylglyoxal (MG) inhibits colon cancer cell proliferation Colon cancer cells were treated with MG at various concentrations (0.4, 0.8, 1.6, or 2.0 mmol/L) or with vehicle alone (0 mmol/L MG). Cell proliferation was determined at 12, 24, 36, and 48 h by the CCK-8 kit. *p < 0.05, ^*^p < 0.01, ^**^*p < 0.001 vs controls (0 mmol/L). All data are representative of three independent experiments (n = 3). Differences between groups were analyzed by analysis of variance. Cell relative activity = {ODsample – ODblank}/{ODcontrol (0 mmol/L) – ODblank}

**Figure 4:**
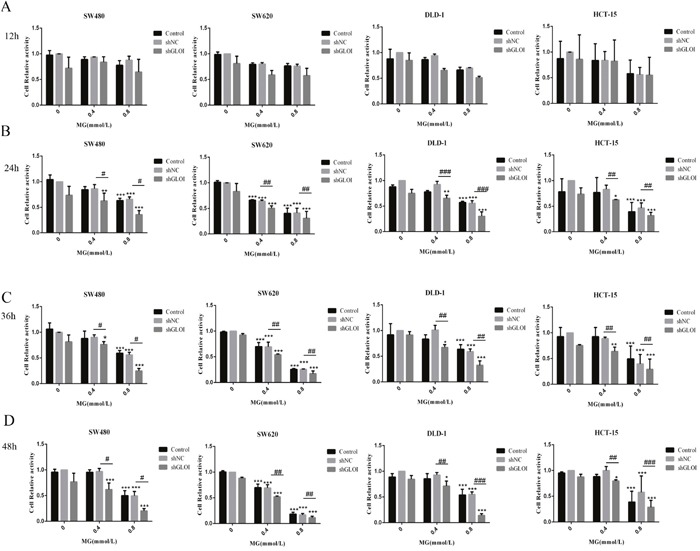
Methylglyoxal (MG), alone or in combination with GLOI silencing, inhibits viability and proliferation of colon cancer cells Colon cancer cells transfected with shGLOI, empty vector (shNC), or nothing, were treated with MG at the doses shown or vehicle alone. The viability of the cells was determined at 12 h **(A)**, 24 h **(B)**, 36 h **(C)**, and 48 h **(D)**. *p < 0.05, ^*^p < 0.01, ^**^*p < 0.001 vs shNC (0 mmol/L); ^#^*p* < 0.05, ^##^*p* < 0.01, ^###^*p* < 0.001 shNC vs shGLOI. All data are representative of three independent experiments (n = 3). Differences between groups were analyzed by analysis of variance. Cell relative activity = {ODsample – ODblank}/{ODshNC (0 mmol/L) – ODblank}

Colon cancer cell viability was inhibited by MG, and the degree of inhibition was dependent on concentration and treatment time (Figure 3). Incubation with MG (0.4 or 0.8 mmol/L), alone or in combination with GLOI silencing, had no notable effect on the viability of SW480, SW620, DLD-1, or HCT-15 colon cancer cells at 12 h (Figure 4A). MG (0.4 mmol/L) or GLOI silencing alone had no notable effect on the viability of SW480, DLD-1, or HCT-15 colon cancer cells at 24 h, 36 h, and 48 h, but the combination did reduce the viability of all three cell types, SW480 (*p* < 0.001), DLD-1 (*p* < 0.001), and HCT-15 (*p* < 0.05), at 24h, 36h, and 48h (Figure 4B-4D). In contrast, MG (0.4 mmol/L) alone or in combination with GLOI silencing inhibited the viability of SW620 cells at 24h, 36h, and 48h (all, *p* < 0.001; Figure 4B-4D). The viability of all four types of colon cancer cells was significantly reduced by treatment with the higher dose of MG (0.8 mmol/L) for 24 h, 36 h, or 48 h, and this inhibitory effect became more pronounced when MG was combined with GLOI silencing (*p* < 0.001; Figure 4B-4D).

**Figure 5:**
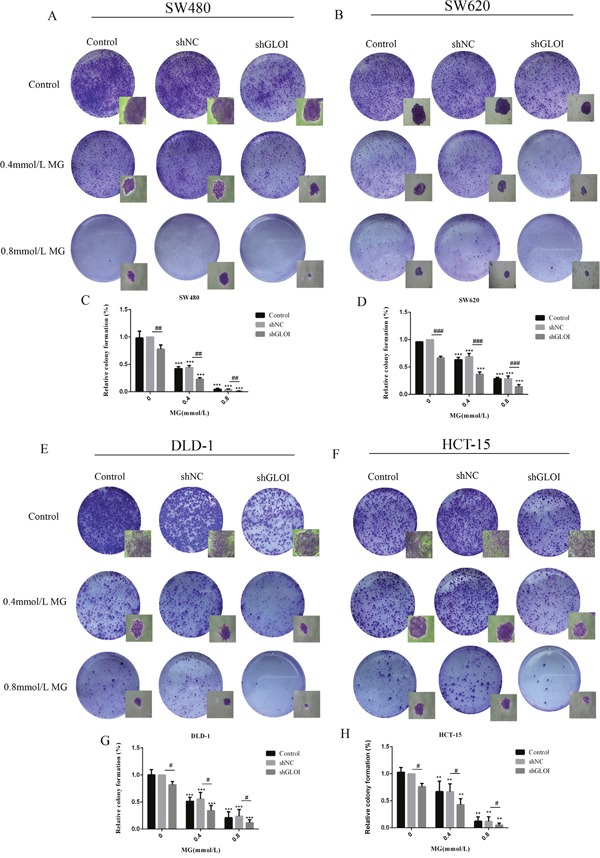
Methylglyoxal (MG), alone or in combination with GLOI silencing, inhibits colony formation by colon cancer cells SW480, SW620, DLD-1, and HCT-15 colon cancer cells transfected with shGLOI, empty vector (shNC, negative control), or nothing (Control), were treated with MG at the concentrations shown or with vehicle only (negative control). Colony formation by the cells treated with MG was significantly suppressed at 10 days. The images **(A, B, E, F)** and the graphs **(C, D, G, H)** as panels were shown. ^*^p < 0.01, ^**^*p < 0.001 vs shNC (0 mmol/L); ^#^*p* < 0.05, ^##^*p* < 0.01, ^###^*p* < 0.001 shNC vs shGLOI. All data are representative of three independent experiments (n = 3). Differences between groups were analyzed by analysis of variance.

Cancer cells treated with MG (0.4 mmol/L) or GLOI silencing alone formed fewer colonies than the controls (*p* < 0.05 to 0.001; Figure 5). There was almost no colony formation by cells treated with the higher dose of MG (0.8 mmol/L; *p* < 0.001; Figure 5). This inhibitory effect on colony formation was even greater in cells treated with combined MG and GLOI silencing (*p* < 0.001; Figure 5).

### MG, alone or in combination with GLOI silencing, inhibited migration and invasion of colon cancer cells

**Figure 6:**
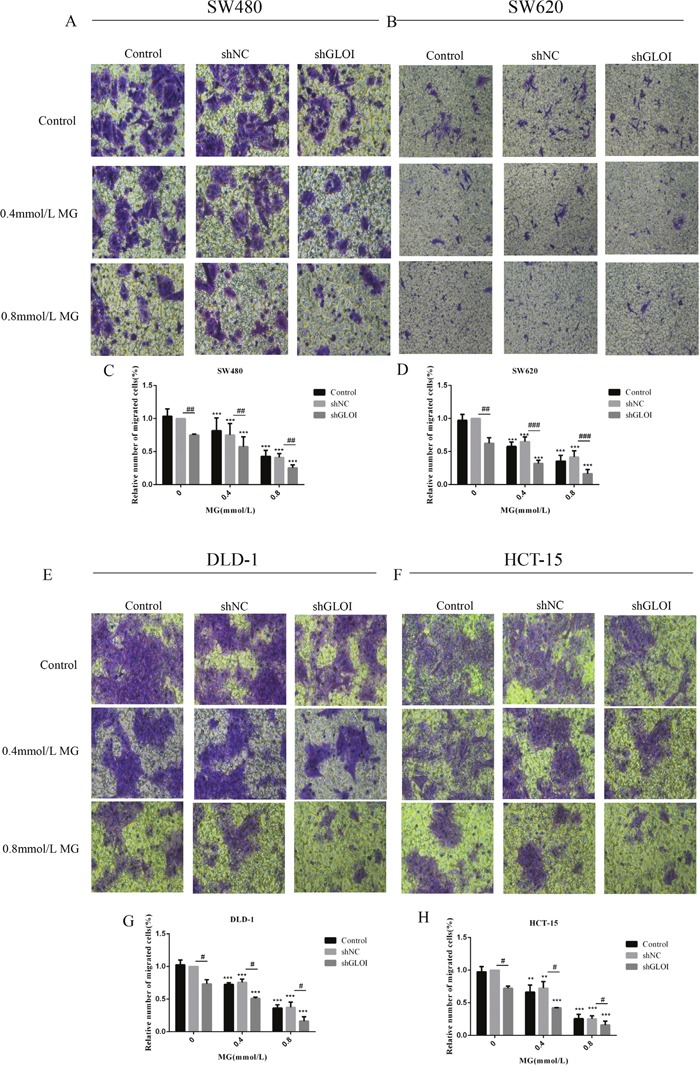
Methylglyoxal (MG), alone or in combination with GLOI silencing, inhibits migration of colon cancer cells SW480, SW620, DLD-1, and HCT-15 colon cancer cells transfected with shGLOI, empty vector (shNC, negative control), or nothing (control), were treated with MG at the concentrations shown or with vehicle only (negative control). Migration of colon cancer cells treated with MG was significantly suppressed, as evaluated by penetration of a transwell insert membrane. The images **(A, B, E, F)** and the graphs **(C, D, G, H)** as panels were shown. ^*^p < 0.01, ^**^*p < 0.001 vs shNC (0 mmol/L); ^#^*p* < 0.05, ^##^*p* < 0.01, ^###^*p* < 0.001 shNC vs shGLOI. All data are representative of three independent experiments (n = 3). Differences between groups were analyzed by analysis of variance.

**Figure 7:**
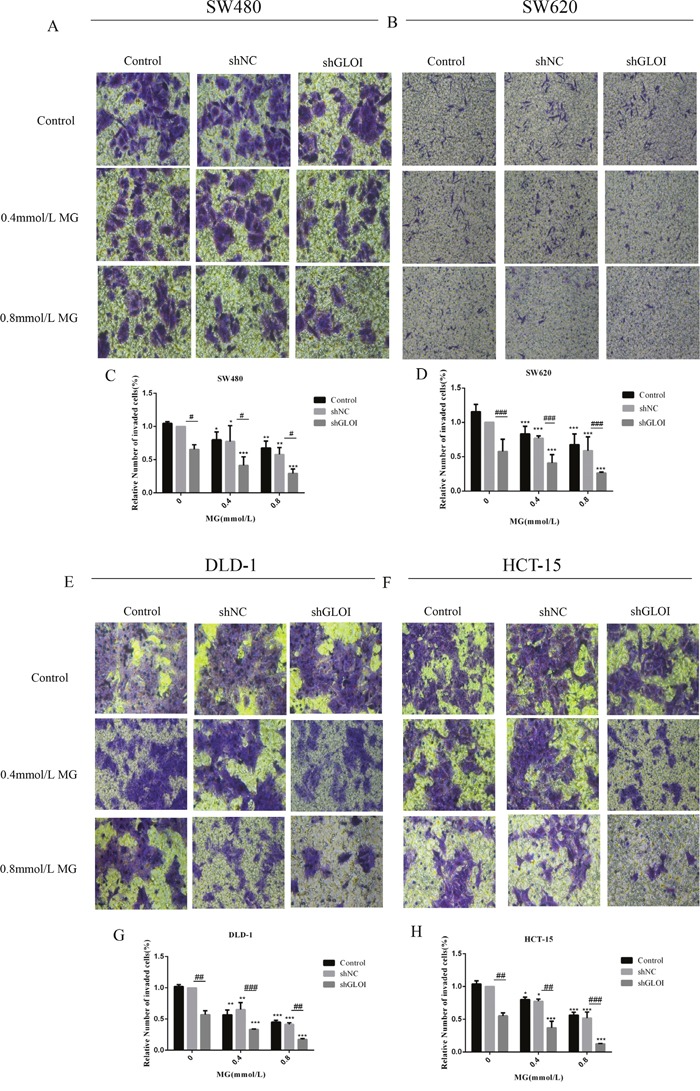
Methylglyoxal (MG), alone or in combination with GLOI silencing, inhibits invasion of colon cancer cells SW480, SW620, DLD-1, and HCT-15 colon cancer cells transfected with shGLOI, empty vector (shNC, negative control), or nothing (Control), were treated with MG at the concentrations shown or with vehicle only (negative control). Invasion of colon cancer cells treated with MG was significantly suppressed, as evaluated by penetration of a matrigel-coated transwell insert membrane. The images **(A, B, E, F)** and the graphs **(C, D, G, H)** as panels were shown. *p < 0.05, ^*^p < 0.01, ^**^*p < 0.001 vs shNC (0 mmol/L); ^#^*p* < 0.05, ^##^*p* < 0.01, ^###^*p* < 0.001 shNC vs shGLOI. All data are representative of three independent experiments (n = 3). Differences between groups were analyzed by analysis of variance.

The number of colon cancer cells penetrating the transwell membrane was lower for the cells treated with MG (0.4 or 0.8mmol/L) or GLOI silencing than for the control cells (*p* < 0.05 to 0.001; Figure 6). This inhibitory effect on migration was even greater when cells were treated with combined MG and GLOI silencing (*p* < 0.05 to 0.001; Figure 6). Similarly, the invasion capacity of the cancer cells was also reduced by MG or GLOI silencing. Treatment with MG (0.4 or 0.8 mmol/L) or GLOI silencing significantly reduced the number of colon cancer cells that invaded through the transwell insert membrane compared to the control cells (*p* < 0.05 to 0.001; Figure 7). Co-treatment with MG and GLOI silencing reduced colon cancer cells invasion to a dramatically greater extent than either treatment alone (*p* < 0.05 to 0.001; Figure 7).

### MG, alone or in combination with GLOI silencing, induced apoptosis in colon cancer cells

**Figure 8:**
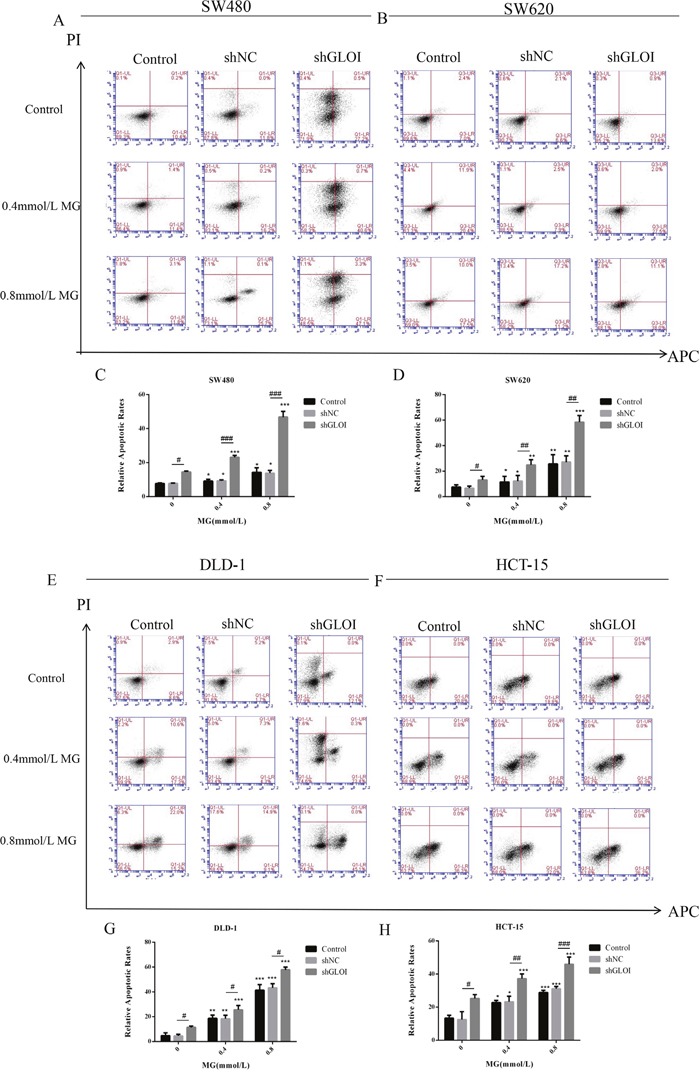
Methylglyoxal (MG), alone or in combination with GLOI silencing, induces apoptosis of colon cancer cells SW480 (A, C), SW620 (B, D), DLD-1 (E, G), and HCT-15 (F, H) colon cancer cells were transfected with shGLOI, empty vector (shNC, negative control), or nothing (Control), were treated with MG at the concentrations shown or with vehicle only (negative control). After 48 h, apoptosis was detected by Annexin V-APC assay and propidium iodide staining. **(A, B, E, F)** Apoptotic cells, including early apoptosis (LR) and late apoptosis (UR), were identified by flow cytometry. **(C, D, G, H)** Histograms show quantification of apoptotic cells. *p < 0.05, ^*^p < 0.01, ^**^*p < 0.001 vs shNC (0 mmol/L); ^#^*p* < 0.05, ^##^*p* < 0.01, ^###^*p* < 0.001 shNC vs shGLOI. All data are representative of three independent experiments (n = 3). Differences between groups were analyzed by analysis of variance.

The apoptosis rate of SW480 cells treated with MG increased slightly over that of untreated cells, to 12.8% (0.4 mmol/L MG) or 14.9% (0.8 mmol/L MG). Co-treatment with MG (0.4 or 0.8 mmol/L) and GLOI silencing increased their apoptosis rate to 41.3% and 50.4% increase compared to shNC-transfected cells, respectively (Figure 8A). The rates for MG (0.4 or 0.8 mmol/L) with shGLOI - transfected cells were 24.9% and 24.6% higher than that for the MG (0.4 or 0.8 mmol/L) with shNC - transfected cells, respectively. Similar effects were observed in SW620, DLD-1, and HCT-15 cells (Figure 8B, 8D-8H). The combination of MG (0.4 or 0.8 mmol/L) with GLOI silencing increased cell apoptosis to a greater degree than either treatment alone (*p* < 0.05 to 0.001; Figure 8).

### MG, alone or in combination with GLOI silencing, up-regulated STAT1 and Bax and down-regulated Bcl-2 in colon cancer cells

**Figure 9:**
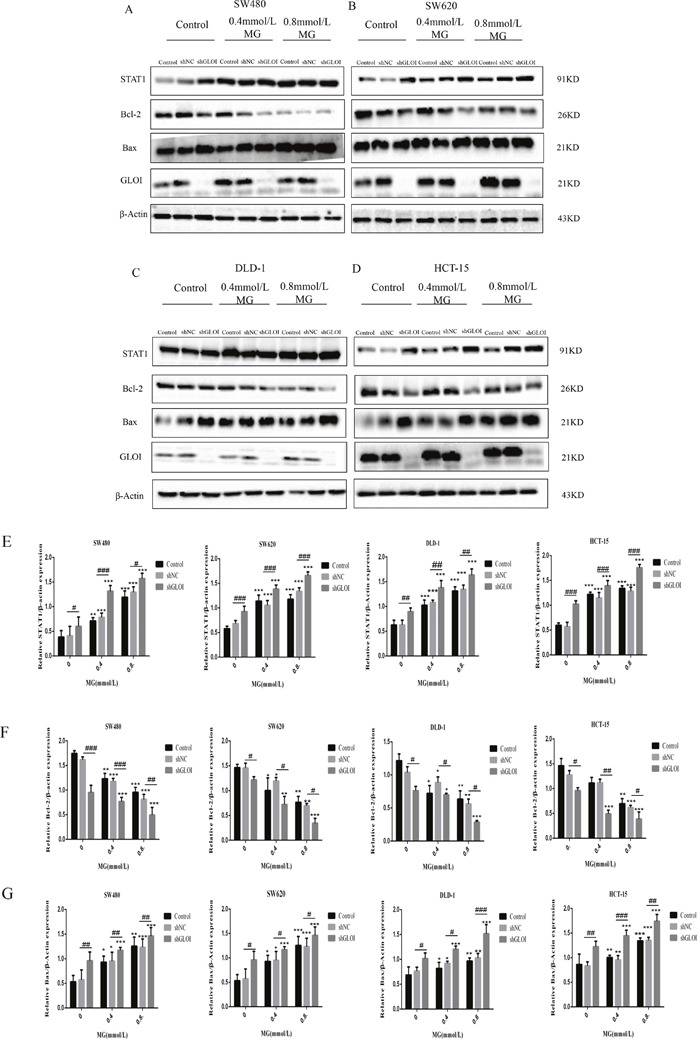
Methylglyoxal (MG), alone or in combination with GLOI silencing, alters the expression levels of STAT1 signal pathway proteins in colon cancer cells Colon cancer cells were transfected with shGLOI, with empty vector (shNC, negative control), or nothing (Control), were treated with MG at the concentrations shown or with vehicle alone (negative control). **(A-D)** Whole-cell levels of STAT1, Bax, and Bcl-2 were analyzed by Western blot. **(E-G)** Protein levels were quantified and graphed. *p< 0.05, ^*^p < 0.01, ^**^*p < 0.001 vs shNC (0 mmol/L); ^#^*p* < 0.05, ^##^*p* < 0.01, ^###^*p* < 0.001 shNC vs shGLOI. All data are representative of three independent experiments (n = 3). Differences between groups were analyzed by analysis of variance.

STAT1 protein levels were increased in all four types of colon cancer cells after treatment with MG (0.4 or 0.8 mmol/L) or GLOI silencing (Figure 9A-9D). Co-treatment with MG (0.4 or 0.8 mmol/L) and GLOI silencing increased STAT1 protein levels by 2-fold and 3-fold in SW480, 3-fold and 4-fold in SW620, 2.5-fold and 3.6-fold in DLD-1, and 2.7-fold and 4-fold in HCT-15 cells, compared to the shNC-transfected cells (Figure 9A-9D). Similarly, Bax was increased in the colon cancer cells under the same conditions (*p* < 0.05 to 0.001; Figure 9A-9D, 9G). In contrast, Bcl-2 levels were decreased by approximately 12%, 51% and 22% when treated with MG (0.4 or 0.8 mmol/L) or GLOI silencing alone compared to the control in HCT-15 cells; after co-treatment with MG (0.4 or 0.8 mmol/L) and GLOI silencing, it decreased by 64% and 80%, respectively, compared to the shNC-transfected cells (Figure 9D). The combination of MG (0.4 or 0.8 mmol/L) with GLOI silencing increased STAT1 and Bax protein levels and decreased Bcl-2 protein levels to a greater degree than either treatment alone (*p* < 0.05 to 0.001; Figure 9).

### MG, alone or in combination with GLOI silencing, increased the intracellular concentrations of MG in colon cancer cells

**Figure 10:**
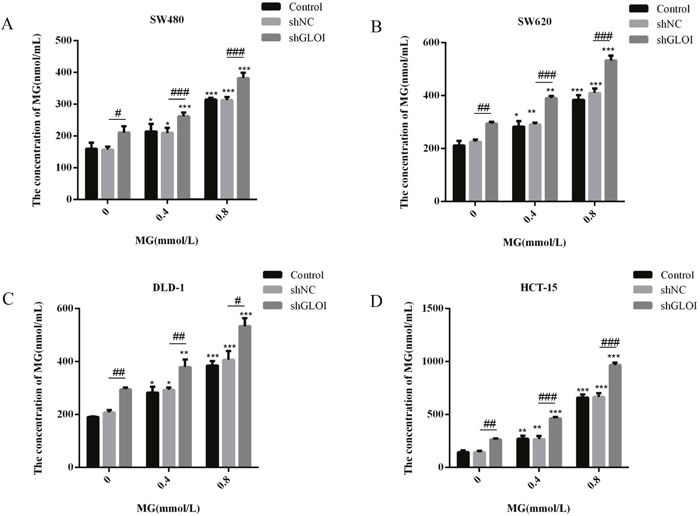
Methylglyoxal (MG), alone or in combination with GLOI silencing, increases the concentration of intracellular MG in colon cancer cells Colon cancer cells were transfected with shGLOI, with empty vector (shNC, negative control), or nothing (Control), were treated with MG at the concentrations shown or with vehicle alone (negative control). Intracellular MG was quantified with an enzyme-based immunoassay after 48 h. *p < 0.05, ^*^p < 0.01, ^**^*p < 0.001 vs shNC (0 mmol/L); ^#^*p* < 0.05, ^##^*p* < 0.01, ^###^*p* < 0.001 shNC vs shGLOI. All data are representative of three independent experiments (n = 3). Differences between groups were analyzed by analysis of variance.

Treatment with MG or GLOI silencing increased the intracellular concentrations of MG in colon cancer cells, with the highest increase observed in HCT-15 cells (Figure 10). When treated with MG (0.4 or 0.8 mmol/L) or GLOI silencing alone, the intracellular concentration of MG reached 2-fold, 5-fold, and 1.5-fold in HCT-15 cells compared to that in shNC-transfected cells (*p* < 0.01 to 001; Figure 10D). The combination of MG (0.4 or 0.8 mmol/L) with GLOI silencing increased intracellular MG concentration by 3.5-fold, 7-fold in HCT-15 cells, compared to that in shNC-transfected cells (Figure 10D). The MG concentration for MG (0.4 or 0.8 mmol/L) with shGLOI - transfected cells were 1.6-fold and 1.4-fold higher than that for the MG (0.4 or 0.8 mM) with shNC - transfected cells, respectively (Figure 10D). The combination of MG (0.4 or 0.8 mmol/L) with GLOI silencing in colon cancer cells increased the intracellular concentration of MG to a greater degree than either treatment alone (*p* < 0.05 to 001; Figure 10).

### MG, alone or in combination with GLOI silencing, reduced tumor growth in BALB/c nude mice

**Figure 11:**
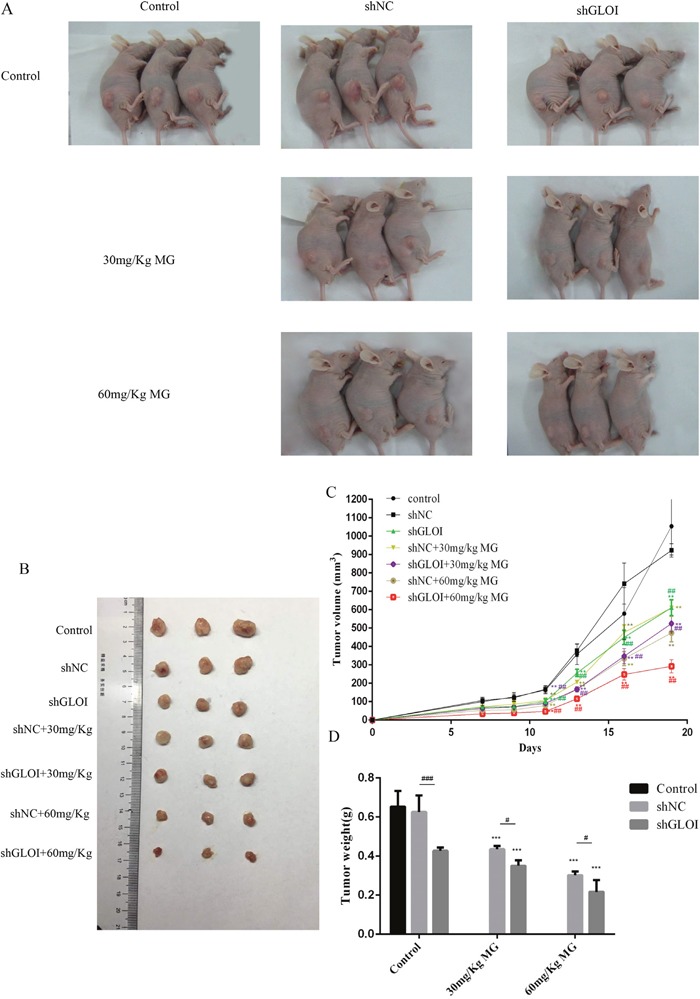
Methylglyoxal (MG), alone or in combination with GLOI silencing, reduces SW620 tumor growth in BALB/c nude mice Xenograft tumors were produced by subcutaneous inoculation of SW620 cells transfected with shGLOI, empty vector (shNC, negative control), or nothing (Control). Seven days later, mice began treatment with MG (30 or 60 mg/kg) or vehicle only (negative control) via intraperitoneal injection every other day. Treatment was continued for 12 days; mice were killed on day 19 and their tumors excised. **(A)** Photographs of representative tumor-bearing mice by treatment group. **(B)** Photographs of representative excised tumors by treatment group. **(C)** Xenograft tumors were measured every 2-3 days during treatment and their volumes calculated. **(D)** The weights of excised tumors by treatment group are shown. All data are representative of three independent experiments (n = 3). ^*^p < 0.01, ^**^*p < 0.001 vs shNC (0 mmol/L); ^#^*p* < 0.05, ^##^*p* < 0.01 shNC vs shGLOI. Differences between groups were analyzed by analysis of variance.

**Figure 12:**
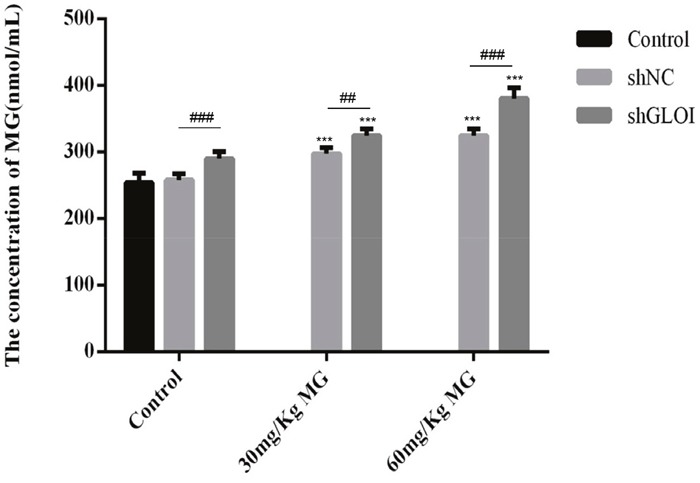
Methylglyoxal (MG), alone or in combination with GLOI silencing, increases the concentration of MG in peripheral blood of mice bearing colon cancer xenografts SW620 xenograft tumors were established in athymic BALB/c nude mice as described in the legend to Figure 11. Beginning on day 7 after tumor cell inoculation, mice were treated with MG at the concentrations shown or with vehicle alone (negative control). MG level in the peripheral blood was measured on day 19 after inoculation of SW620 cells by an enzyme-based immunoassay. ^**^*p < 0.001 vs shNC (0 mmol/L); ^##^*p* < 0.01, ^###^*p* < 0.001 shNC vs shGLOI. All data are representative of three independent experiments (n = 3). Differences between groups were analyzed by analysis of variance.

Significant inhibition of SW620-induced tumor growth was observed on day 14 after injection of SW620 cells (treatment day 7) in mice treated with MG alone or in combination with GLOI silencing (*p* < 0.05 to 0.001; Figure 11A). Administration of MG at doses of 30 mg/kg or 60 mg/kg reduced the tumor weights by 40% and 60%, respectively, by day 19, whereas the combination of MG treatment with GLOI silencing in habited tumor weights even further, by 60% and 70%, respectively (*p* < 0.05 to 0.001; Figure 11B, 11D). Treatment with MG, GLOI silencing, or the combination increased the concentration of MG in the peripheral blood of the treated mice (*p* < 0.05 to 0.001; Figure 12).

**Figure 13:**
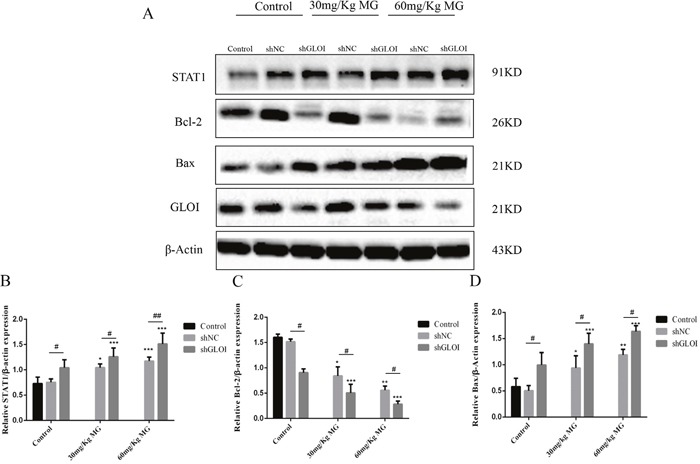
Methylglyoxal (MG), alone or in combination with GLOI silencing, affects expression of STAT1 signal pathway proteins in a colon cancer mouse model *in vivo* SW620 colon cancer xenografts were established as described in the legend to Figure 11. Beginning on day 7 after tumor cell inoculation, mice were treated with MG at the concentrations shown or with vehicle alone (negative control). **(A)** Expression levels of STAT1 signal pathway proteins STAT1, Bcl-2, and Bax and of GLOI were analyzed by Western blot. Treatment with MG upregulated STAT1 and Bax but downregulated Bcl-2. **(B-D)** Protein levels were quantified and graphed. *p < 0.05, ^*^p < 0.01, ^**^*p < 0.001 vs shNC (0 mmol/L); ^#^*p* < 0.05, ^##^*p* < 0.01 shNC vs shGLOI. All data are representative of three independent experiments (n = 3). Differences between groups were analyzed by analysis of variance.

**Figure 14:**
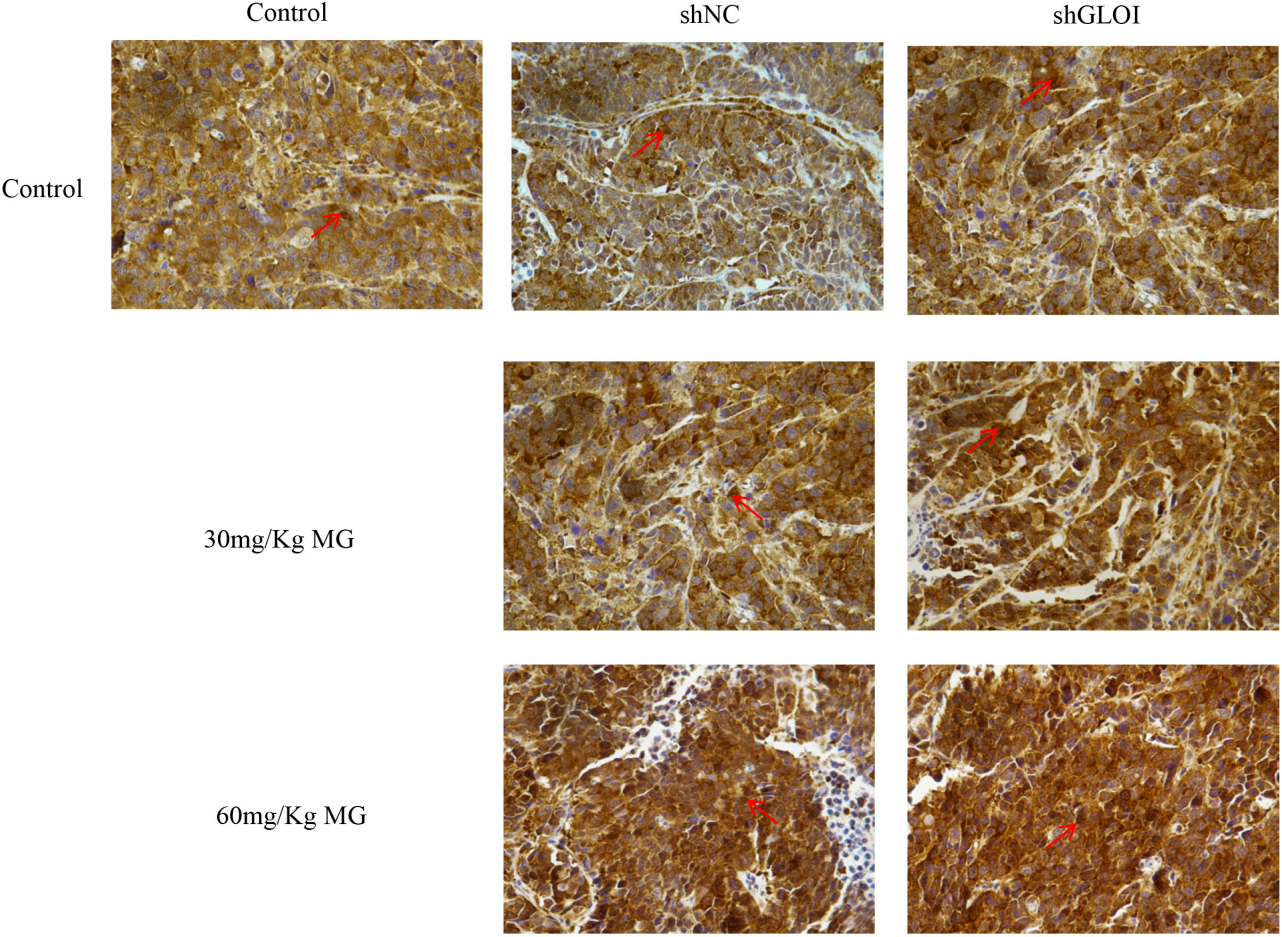
Methylglyoxal (MG) upregulates expression of STAT1 in a colon cancer xenograft model SW620 colon cancer xenografts were established as described in the legend to Figure 11. Beginning on day 7 after tumor cell inoculation, mice were treated with MG at the concentrations shown or with vehicle alone (negative control). The expression of STAT1 in the excised tumors was determined by immunohistochemical staining; representative images are shown (original magnification, ×400). The arrows are pointing at STAT1 positive cells.

STAT1 protein levels in tumor tissues were increased to approximately 1.3-fold in mice treated with 30 mg/Kg MG and 1.7-fold in mice treated with 60 mg/Kg MG relative to that in the tumors of the control group (Figure 13A). However, STAT1 levels increased 2-fold (30 mg/kg) and 2.8-fold (60 mg/kg) in the tumors of mice treated with the combination of MG and GLOI silencing. This result was confirmed by immunohistochemical analysis, which showed that STAT1 expression was higher in tumors from mice treated with MG than in tumors from the controls (Figure 14). Similarly, MG treatment up-regulated Bax and down-regulated Bcl-2, and MG combined with GLOI silencing augmented these effects (*p* < 0.05 to 0.001; Figure 13A, 13C, 13D).

## DISCUSSION

Our findings demonstrate that MG treatment, GLOI silencing, or combination of the two suppressed the viability, proliferation, migration, and invasion of SW480, SW620, DLD-1, and HCT-15 colon cancer cells and increased their apoptosis *in vitro*. These treatments also increased expression of STAT1 and Bax and decreased expression of Bcl-2 in these cells. Furthermore, treatment with MG with or without GLOI silencing inhibited SW620 tumor growth *in vivo* in a mouse xenograft model. As in the *in vitro* model, treatment with MG with or without GLOI silencing increased expression of STAT1 and Bax in the tumors from these mice and decreased expression of Bcl-2. The concentrations of MG in the colon cancer cells and in the blood of the mice were increased following treatment with MG, GLOI silencing, or the combination.

Our group and others have shown the antitumor efficacy of MG [12–14, 32–34]. Monika et al. showed that MG inhibited proliferation of human glioblastoma multiforme cells [14]. Bair et al. showed that MG exerted antiproliferative activity in human malignant melanoma [19]. Another study demonstrated that MG inhibited PC-3 prostate cancer cell growth [35]. GLOI has been shown to be overexpressed in numerous tumor tissues and cells, including colon, breast, prostate, stomach, pancreatic, and kidney cancers, while depletion of GLOI expression significantly inhibited the proliferation of these tumor cells [20, 22, 24, 36]. Inhibition of GLOI sensitized human melanoma cells to the well-established antiproliferative, apoptotic, and oxidative stress–inducing activity of exogenous MG [19]. Our results showing that MG, alone or in combination with GLOI silencing, inhibited the viability and proliferation of colon cancer cells are in accordance with published results in other cancer types. And our study also demonstrates that combination of MG with GLOI silencing exerts synergistic inhibitory effects on colon cancer cells’ viability and proliferation. This synergistic effect may be attributed to that GLOI silencing not only increased the concentration of MG [37] but also increased the sensitivity of cells to MG. Another study also indicated that GLOI silencing could improve the sensitivity of cells to chemotherapeutic drugs [25].

Lorena et al. showed that MG down-regulated migration and invasion of liver cancer cells and that these effects were p53-dependent [32]. The down-regulation of migration and invasion induced by GLOI depletion was observed in gastric cancer cells [38]. Our group showed that MG treatment and GLOI silencing reduced the migration and invasion of breast cancer cells [12]. These findings support our results showing that MG treatment or GLOI silencing markedly reduces the migration and invasion of colon cancer cells.

In this study, significantly increased apoptosis in association with up-regulation of Bax and down-regulation of Bcl-2 were observed in colon cancer cells treated with MG or GLOI silencing. Kang et al. reported that MG triggered apoptosis in neurons by down-regulating Bcl-2 and up-regulating Bax [39]. MG triggered apoptosis in several malignant cell types, including HeLa, leukemia 60, and prostate cancer cells [35, 40, 41]. Another study showed that GLOI inhibition or treatment with MG enhanced apoptosis induced by TRAIL via down-regulation of Bcl-2 expression [42].

The up-regulation of STAT1 in our study by treatment with MG, alone or combination with GLOI silencing, is interesting because STAT1 is a known tumor suppressor [27, 29]. STAT1 is involved in defense against pathogens and the inhibition of cell proliferation in SW480 colon cancer cell [43]. In addition, STAT1 has been shown to enhance the transcriptional activity of p53 on several p53-responsive pro-apoptotic genes such as Bax and to negatively regulate the Bcl-xL promoter [27, 28, 44]. Studies have shown that MG markedly increased IFN-γ in Sarcoma180 tumor-bearing mice [45], and STAT1 can be up-regulated by IFN-γ [46]. Our results suggest that STAT1, Bax, and Bcl-2 could be involved in the antiproliferative and pro-apoptotic effects of MG and silencing of GLOI in colon cancer cells. However, the exact molecular mechanisms underlying the regulation of these proteins and their relationships with each other need further investigation.

Early study demonstrated that MG decreased in cancer and GLOI activity increased in colon cancer cells compared to the normal cells [22, 47]. A literature demonstrated that silencing of GLOI increased the concentration of MG in L6 Myoblasts [25]. Our results suggest that the concentration of MG increased after MG treatment or GLOI silencing.

In summary, our study results clearly demonstrate that treatment with MG or silencing of GLOI can restrain proliferation, migration, and invasion and induce apoptosis in human colon cancer cells. These effects may be modulated by up-regulation of STAT1 and Bax and down-regulation of Bcl-2. Moreover, treatment with MG, alone or in combination with GLOI silencing, suppressed the growth of SW620-induced tumors in BALB/c nude mice *in vivo*. Combination of MG with GLOI silencing exerts synergistic inhibitory effects on colon tumorigenesis and colon cancer growth mediated through up-regulation of STAT1 and Bax and down-regulation of Bcl-2. These findings suggest a promising therapeutic approach against colon cancer.

## MATERIALS AND METHODS

### Cell lines and culture

Human colon cell lines SW480 (Dukes type B), SW620 (Dukes type C), DLD-1 (Dukes type C), and HCT-15 (Dukes type C), representing different pathological stages of colon cancer, were purchased from the Institute of Biochemistry and Cell Biology, Chinese Academy of Sciences (Shanghai, China). SW480 was established from a primary adenocarcinoma of the colon and SW620 established from a lymph node metastasis taken from the same patient one year later is available. DLD-1 and HCT-15 (CCL-225) were independently derived from different clonal origin from the same individual. Normal FHC colon cells were obtained from Key Laboratory of Laboratory Medicine, Ministry of Education of China, Zhejiang Provincial Key Laboratory of Medical Genetics, School of Laboratory Medicine and Life Sciences, Wenzhou Medical University, Wenzhou, Zhejiang 325035, China. The cells were cultured in RPMI-1640 medium supplemented with 10% fetal bovine serum (FBS; Bioind, Beit-Haemek, Israel) and 100 U/mL penicillin-streptomycin (P1400; Solarbio, Beijing, China) at 37°C in an incubator containing a humidified atmosphere supplemented with 5% CO_2_. All four cell types were used in all experiments.

### Plasmid transfection

The GLOI shRNA targeting sequences (shGLOI) and empty vector (shNC, negative control) were synthesized by GenePharma (Shanghai, China). The group in which cells received no transfection was defined as Control. Cells were grown to 80-90% confluence, then transfected with one of these constructs. Transfection was carried out with Lipofectamine 2000 (Invitrogen, Shanghai, China; DNA: Lipofectamine 2000 ratio, 4:5) according to the manufacturer's instructions. After transfection for 6 h, the culture medium was replaced with fresh RPMI-1640 containing 10% FBS. The fluorescence comes from sh-vectors pGPU6/GFP/Neo. According to the manufacturer's instruction (GenePharma), transfection efficiency is directly correlated with fluorescence determined by fluorescence (green) microscope. The transfection efficiency of cells= cells of the green fluorescent protein/total number of cells ×100 %. Stable transfectants (transfection efficiency ≥ 50%) were established by incubating cells in complete RPMI-1640 medium with 1000 μg/mL G418 (11811031; Sigma, St Louis, MO) for 15 days. The clones were verified by Western blot, real-time quantitative reverse-transcription polymerase chain reaction (RT-PCR), and quantification of GLOI enzymatic activity. The successful clones were pooled and used for further experiments. The sequences of shGLOI were GGATTCGGTCATATTGGAATT; GGGAGTCAAATTTGTGAAGAA; GGCATTTATTCAA GATCCTGA; and GAAGAACTGGGAGTCAAATTT.

### Quantification of GLOI mRNA

Total RNA was extracted from cells or dissected tumors by using TRIzol (3101-100; Invitrogen) according to the manufacturer's instructions. Total RNA was reverse-transcribed to cDNA, which was evaluated by using an Applied Biosystems 7500 Fast Sequence Detection System and SYBR Green PCR Kit (20454; Qiagen, Shanghai, China) under the following conditions: denaturation at 95°C for 5 min, followed by 40 cycles of denaturation at 95°C for 10 s and annealing and extension at 60°C for 30 s. The GLOI mRNA expression was normalized to that of GAPDH. Random primer (RRO37A; Takara, Shiga, Japan). Primers (Sangon Biotech, Shanghai, China) for GLOI were 5′-AGCAGACCATGCTACGAGTG-3′ (forward) and 5′-TAGCTTTTCTGGAGAGCGCC-3′ (reverse). Primers for the internal control GAPDH were5′-AAGGTGAAGGTCGGAGTCAAC-3′ (forward) and 5′-GGGGTCATTGATGGCAACAATA-3′ (reverse).

### Quantification of GLOI enzymatic activity

GLOI enzyme activity was determined using the QuantiChrom Glyoxalase I Assay Kit (Shanghai Universal Biotech Co., Shanghai, China), following manufacture's instruction. In brief, cells transfected with shGLOI or shNC were trypsinized and collected by centrifugation at 650g for 5 min. After two washes with phosphate-buffered saline solution (PBS), the treated cells were subjected to lysis in the presence of a protease inhibitor cocktail and then centrifuged at 12,000g for 15 min at 4°C. The supernatant concentration was measured by using a bicinchoninic acid protein assay kit (P0010; Beyotime, Hangzhou, China). The working liquid and blank control were prepared and centrifuged. The supernatant fraction (200 μL) was placed in 96-well culture plates. Absorbance was measured at 240 nm using a Multiskan Spectrum reader (Thermo Scientific, Waltham, MA, USA). GLOI enzymatic activity =175×(ODsample-ODblank) [12].

### Cell viability assay

Effects on cell viability were determined with the Cell Counting Kit 8 (CCK-8) assay (CK04; Dojindo, Kumamoto, Japan). Details of this assay have been described elsewhere [45]. In short, cells were seeded in 96-well culture plates (3×10^3^ cell/well, SW480 and DLD-1; 2.5×10^3^ cell/well, SW620; 2×10^3^ cell/well, HCT-15) and allowed to adhere for 12 h. The cells were treated with one of two concentrations of MG (0.4 or 0.8 mmol/L) or control vehicle for 12, 24, 36, or 48 h. The CCK-8 assay reagent (10 μL) was added to each well, and the plates were incubated at 37°C for 1 h. Absorbance was measured at 450 nm using an Multiskan Spectrum reader (Thermo Scientific, Waltham, MA, USA).

### Colony formation assay

The culture plate colony formation assay was used to detect cell proliferation [45]. Cells were seeded in 6-well culture plates (3×10^3^ cells/well) and treated with MG (0.4 or 0.8 mmol/L) or vehicle and/or shGLOI to silence GLOI. After 10 days, the resulting colonies were disposed and fixed with 4% cold paraformaldehyde for 15 min and stained with crystal violet (C0121; Beyotime, Hangzhou, China) for 15 min at room temperature. After two washings with PBS, the colonies were viewed and counted under a microscope at ×40 magnification. Only clearly visible colonies (diameter > 50 μm) were counted.

### Transwell assays

Cell migration and invasion were assessed by transwell assays. Transwell plates with an 8-μm pore membrane insert (Corning, Shanghai, China) were used. For the migration assay, cells were seeded in 6-well culture plates (3×10^3^ cells/well), after treatment colon cancer cells with MG for 24 h, cells were collected and washed twice with serum-free RPMI-1640 medium and resuspended in the same medium. The cells were seeded into the upper chambers of the transwell culture plates (SW480, 8×10^4^ cells; SW620, 1.2×10^5^ cells; DLD-1, 8×10^4^ cells; and HCT-15, 8×10^4^ cells), and RPMI-1640 medium containing 20% FBS was placed in the lower chambers as a chemoattractant. The cells were seeded into the upper chambers of the transwell culture plates as described in our previous paper [13]. The invasion assay was similar to the migration assay except that the inserts were coated with 45 uL matrigel solution (matrigel: serum-free medium ratio 1:10). All the above steps were carried out on the ice. After 48 h, the cells that adhered to the upper surface of the membranes were removed with a cotton swab, and the cells that had penetrated the membrane (and, for the invasion assay, the matrigel) and were attached to its lower surface were fixed with 4% paraformaldehyde for 15 min and stained with crystal violet. These cells were counted in five randomly chosen regions under a microscope (Nikon, Tokyo, Japan) at ×400 magnification.

### Apoptosis assay

The Annexin V-APC Apoptosis Detection Kit (KGA1030; KeyGEN Biotech) was used to identify apoptotic cells as described in our previous studies and others [13, 48]. In brief, the cells were seeded in 6-well plates (6×10^5^ cells/well). After incubation with MG for 24 h, cells were collected and washed twice with PBS. The cell suspensions were stained with annexin V and PI from the kit for 30 min at 4°C in the dark. The apoptotic cells were then quantified by flow cytometry analyzer (BD Biosciences, San Jose, CA). BD Accuri C6 software was used to analyze the data. Cell apoptosis was determined as either annexin positive or both annexin and PI positive by flow cytometry and the percentage of apoptotic cells was calculated.

### Western blotting analysis

Cells or tissues were harvested and subjected to lysis in the presence of a protease inhibitor cocktail. The supernatant was collected and the protein concentration was measured by using a bicinchoninic acid protein assay kit (P0010; Beyotime, Hangzhou, China). Equal amounts of protein sample (20 μg) were subjected to electrophoresis on a polyacrylamide mini-gel with 12% sodium dodecyl sulfate. Proteins were transferred onto a nitrocellulose membrane, and after blocking with 5% nonfat milk for 2 h at room temperature, the membranes were incubated at 4°C overnight with appropriate antibodies. Antibodies used included anti-GLOI antibody (1:1000 dilution; Sangon, shanghai, China), anti-STAT1 antibody (1:1000 dilution; Abcam, always indicate location like state or country), anti-Bcl-2 antibody (1:2000 dilution; Abcam, Cambridge, English), anti-Bax antibody (1:3000 dilution; Abcam, Cambridge, English), and anti-β-actin antibody (1:1000 dilution; Beyotime, Hangzhou, China). Membranes were washed and incubated with corresponding horseradish peroxidase–conjugated secondary antibody (1:2500 dilution; Beyotime, Hangzhou, China) at room temperature. The membranes were then washed with a mixture of tris-buffered saline solution and Tween 20 and incubated with enhanced chemiluminescence solution, and the final signals were tested and quantified by densitometry using Quantity One software (Bio-Rad, Hercules, CA).

### Tumor xenografts in mice

We purchased 42 male athymic BALB/c nude mice (4 weeks old) from the Shanghai Medical Experimental Animal Care Commission (Shanghai, China), sufficient for seven treatment groups of six mice each. All animal procedures and experimental protocols were approved by the Laboratory Animal Ethics Committee of Wenzhou Medical University. Each mouse was injected subcutaneously in the left dorsal flank with SW620 cells (4×10^6^ in 200 uL of medium) stably transfected with shGLOI or shNC or untransfected controls. Seven days later, tumors were measured in two perpendicular axes by vernier caliper, and tumor volumes were calculated using the formula: volume = (length × width^2^)/2. The mice then began receiving intraperitoneal injections of MG (30 or 60 mg/kg) dissolved in physiological saline solution or saline solution alone every 2 days. After 12 days of treatment, significant variations in tumor size among the groups were apparent. The mice were killed by cervical dislocation and the tumors dissected; the tumors were weighed, and portions were fixed in 4% paraformaldehyde or frozen at -80°C for further work.

### Immunohistochemistry

STAT1 expression in tumor tissues was determined by immunohistochemical analysis by using a kit (Boster, Wuhan, China) according to the manufacturer's instructions. In short, the formalin-fixed, paraffin-embedded tumor sections were deparaffinized and rehydrated through a series of ethanol washes. Slides were steamed for 30 min in 1× sodium citrate buffer solution to repair antigens. Tumor sections were probed with the anti-STAT1 antibody (1:3000 dilution in 5% bovine serum albumin) and incubated at 4°C overnight. The sections then were incubated with secondary antibody for 1 h at 37°C. The slides were stained with 3, 3-diaminobenzidine, counterstained with hematoxylin, and photographed under a microscope.

### Measurement of MG level

MG measurement was determined using the ELISA Kit for Human MG (Shanghai Westang Bio-Tech Co., Shanghai, China) following manufacture's instruction. In brief, cells were harvested and subjected to lysis buffer (RIPA to PMSF ratio, 100:1), and then centrifuged at 14,000× g for 15 min at 4 °C. The supernatant fraction was collected and diluted by 10-fold lysis buffer (RIPA to PMSF ratio, 100:1). Standard solution was prepared. Standard or samples (20 μL) and 100 μL enzyme substrate working liquid were added in each well of 96-well plate and incubated at 37°C for 15 min. Absorbance was measured at 550 nm using a Multiskan Spectrum reader (Thermo Scientific, Waltham, MA, USA) within 30 min. All OD value should be deducted from the blank value after calculation. The sample OD value representing MG concentration was calculated against the standard curve.

### Statistical analysis

Results were obtained from at least three independent experiments. All results were expressed as mean ± standard deviation (SD). Differences between groups were analyzed by one-way analysis of variance (ANOVA) with Dunnett's test or 2-tailed Student t-test. A p-value <0.05 was considered statistically significant. Statistical analyses were performed with SPSS 17.0 (Chicago, IL) and GraphPad Prism 5 (La Jolla, CA) software packages.

## Abbreviations

CCK-8: Cell Counting Kit-8
FBS: fetal bovine serum
GLOI: glyoxalaseI
MG: methylglyoxal
PBS: phosphate-buffered saline solution
qRT-PCR: quantitative real-time polymerase chain reaction
STAT1: signal transducer and activator of transcription1
